# Linking farmers’ perceptions and management decision toward sustainable agroecological transition: evidence from rural Tunisia

**DOI:** 10.3389/fnut.2024.1389007

**Published:** 2024-05-13

**Authors:** Asma Souissi, Boubaker Dhehibi, Ali M. Oumer, Rihab Mejri, Aymen Frija, Meriem Zlaoui, Mohamed Zied Dhraief

**Affiliations:** ^1^International Center for Agricultural Research in The Dry Areas (ICARDA), Tunisia, Tunisia; ^2^Institut National de la Recherche Agronomique de Tunisie (INRAT), Ariana, Tunisia

**Keywords:** participatory approach, agroecological transformation, perceptions, resilience, value chain, North Africa

## Abstract

Global food systems face sustainability challenges like undernourishment, inequity, resource degradation, and pollution. Food production and consumption drive environmental change with greenhouse gas emissions, biodiversity loss, and land-system shifts. The climate change crisis has intensified concerns about the ecological impact of these systems. Sustainable food networks, such as community-supported agriculture, are promoting sustainable production and consumption through short supply chains. International bodies like the Food and Agriculture Organization (FAO) and the Consultative Group for International Agricultural Research (CGIAR) are also spearheading initiatives for more equitable and sustainable food systems. In Tunisia, where dryland areas predominate, the ongoing implementation of the Agroecology Initiative provides the context for this study, which explores the drivers and barriers of agroecological transformation in this challenging environment. The research focuses on stakeholder engagement, with a gender perspective to explore farmer perceptions. The study, conducted in the northwest of Tunisia in 2022–2023, involved focus groups, workshops, surveys, and questionnaires with various stakeholders. Findings highlight farmer organizations’ potential in promoting sustainable farming, with clear goals, diversified systems, and collaborations. However, challenges such as input scarcity, water shortage, low income, and marketing must be addressed. Results also indicate that over 90% of farmers who received assistance with agroecological practices reported a change in their ideas and practices. Fifty seven percent of the workshops participants identified the olive oil value chain as having the greatest potential for agroecological transformation, but it faces constraints such as climate, lack of policy incentives, training, funding, and difficulty in adopting technical innovations. Women’s inclusion in agriculture, environmental, social, and economic challenges were also highlighted. Despite these obstacles, key drivers for agroecological transition were identified. These include the compatibility of many agroecological practices with existing farmer capabilities, their cultural and economic benefits, and the positive outcomes for environmental sustainability and health. The study advocates for a socio-technical systems analysis to address the root causes hindering Tunisia’s agroecological transformation. A participatory approach is crucial to understanding priorities and developing a sustainable and resilient food system. Furthermore, the research underscores the importance of considering diverse farmer perspectives and tailoring strategies to support this critical transition effectively.

## 1 Introduction

Global food systems are struggling to achieve sustainable development goals, contributing to undernourishment, inequity, natural resource degradation, and environmental pollution. Current food systems are vulnerable to multiple shocks, such as climate change, economic crises, and pandemics, which can have cascading effects on smallholder food security. The rising prices of fertilizers and food imports resulting from these shocks have rekindled interest in the call for a policy shift toward agroecology (1). Food production and consumption are major contributors to global environmental change, including greenhouse gas emissions, biodiversity loss, and land-system change (2).

Alternative food networks, such as food cooperatives and community-supported agriculture, aim to promote sustainable production and consumption through short supply chains and connections between consumers and producers. These networks also foster social interactions and collective mindfulness for a sustainable food system. Producers face both pressure and opportunities to incorporate sustainability into their business practices to meet consumers’ expectations. The agroecological transition is a promising approach to create more equitable and ecologically sustainable food systems (3). Agroecology is the application of ecological principles to agricultural systems, offering solutions to farming and food security challenges such as drought, hunger, poverty, and inequality (4). It supports small-scale farmers in diversity and ensures a long-term balance between food production and the sustainability of natural and environmental resources. It also transforms food systems and ensures resilience by balancing between socio-economic and environmental facets.

According to Dagunga et al. (5), promoting agroecology in smallholder farming communities faces both challenges and opportunities. Some of the opportunities for promoting agroecology, include the potential for increased productivity, improved soil health, and enhanced biodiversity. However, there are many challenges to this transition, such as institutional, social, technical, economic, and environmental factors. These challenges include limited access to resources, such as land, water, and capital, as well as inadequate policy support and institutional frameworks. Additionally, there may be cultural and social barriers to the adoption of agroecological practices (6). Previous research also highlights the importance of participatory approaches and knowledge sharing in promoting agroecology among smallholder farmers (5).

International bodies like the Food and Agriculture Organization (FAO) and the Consultative Group for International agricultural Research (CGIAR) are introducing initiatives to promote more equitable and ecologically sustainable food systems. The agroecological transformation initiative,^1^ which promotes good governance of natural resources, input reduction and biodiversity, as well as social and cultural inclusion, equity, and knowledge sharing, is seen as an opportunity for a shift toward more sustainable, inclusive, and resilient food systems (7).

This study is part of the “Agroecological Transformation in Food, Land and Water Systems” initiative launched by the CGIAR and implemented in Tunisia by the International Centre for Agricultural Research in the Dry Areas (ICARDA). This research contributes to addressing the climate change crisis and to enhancing the resilience of food systems. This research aims to investigate the barriers of agroecological transformation in the dryland context based on the involvement of the different stakeholders with a special emphasis on farmers’ beliefs, experiences, and characteristics. Farmers, perception is analyzed, considering the gender perspective. Focusing on dryland areas is crucial due to their unique challenges and characteristics such as water scarcity, erratic rainfall, and fragile ecosystems. Contrasting with more temperate or humid regions, the dryland context requires tailored solutions that consider the specific needs and constraints of farmers operating in these environments.

## 2 Conceptual framework

Conventional expert-led change assessment methods based on top-down approaches generate quantifiable indicators that allow regional or national comparisons. However, they have certain shortcomings, such as alienating local communities and failing to capture the views of diverse stakeholders (8). Involving the community in evaluation procedures means that indicators are more relevant and specific to the context and evolve over time with the community. Participation leads to the empowerment and capacity building of communities to address emerging challenges in their local environment (8). The agroecological transition is a process that involves the adoption of innovative practices that aim to balance productivity with environmental protection. These practices require a significant change in the way farmers manage their crops and natural resources. Therefore, the adoption of agroecological innovations is subject to various uncertainties and risks, which can influence farmers’ perceptions of the innovation (9). Perceptions, which refer to individuals’ interpretations and understanding of received information, play a crucial role in the agroecological transition. In this context, farmers’ perceptions of innovation can greatly shape their willingness to adopt it. These perceptions can be influenced by various factors, such as the perceived advantages and drawbacks of the innovation, the compatibility with existing practices, the level of information and experience, and the social and cultural context (10).

**Figure 1:**
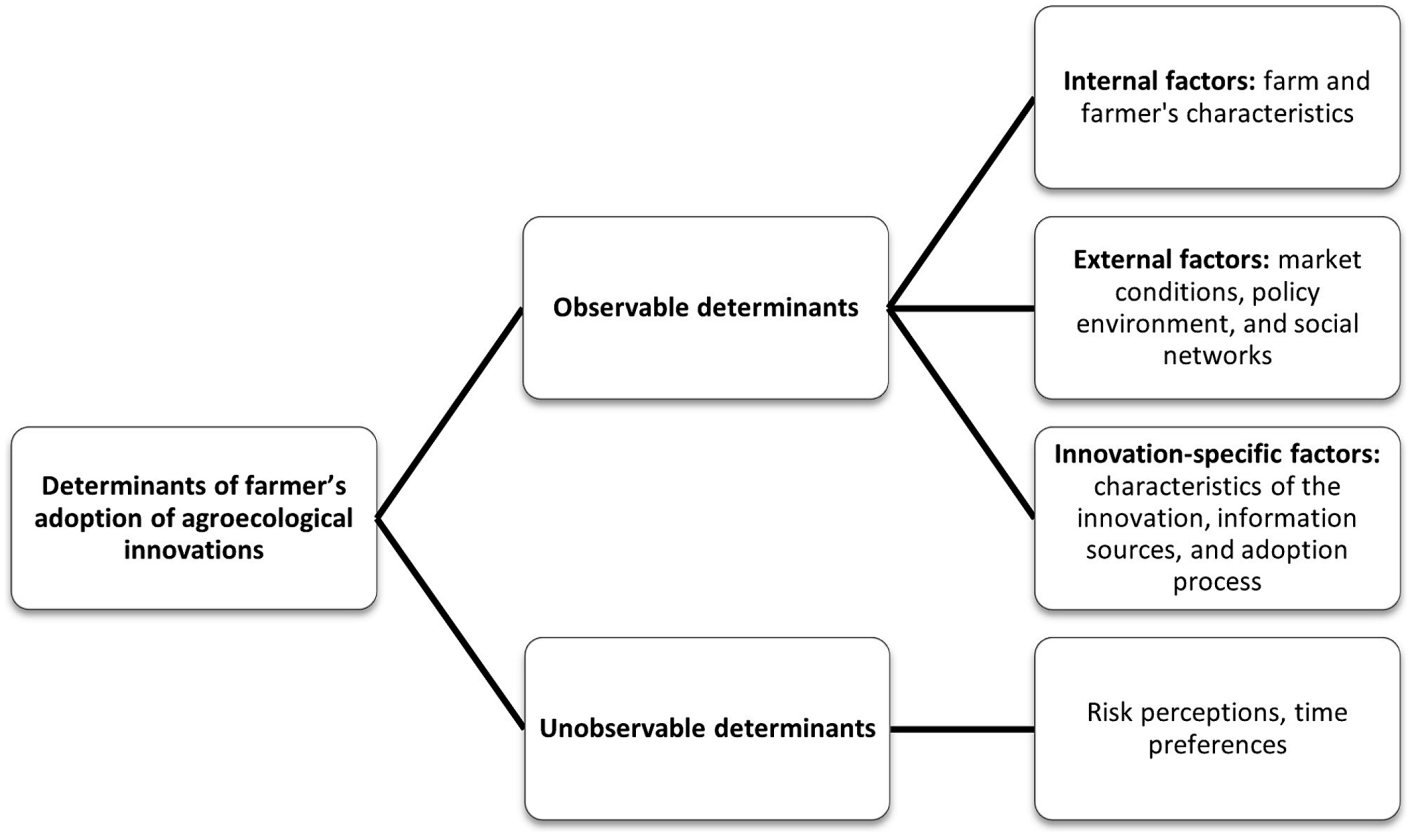
Factors influencing farmers’ adoption of agroecological innovations, adapted from Roussy et al. (12).

To understand the role of perceptions in the agroecological transition, researchers use various experimental methods, such as surveys, interviews, or focus groups. These methods help identify the factors that influence farmers’ perceptions of innovation and how these perceptions impact their decision-making process (11, 12). According to Roussy et al. (12), the factors that influence the adoption of agricultural innovations by farmers are observable and unobservable. Three main categories are identified as observable: external factors, internal factors, and innovation-specific factors. External factors include market conditions, policy environment, and social networks. Internal factors include farmer characteristics, farm characteristics, and risk attitudes. Innovation-specific factors include characteristics of the innovation, information sources, and adoption process (Figure 1).

Considering farmers’ perceptions of these factors in the agroecological transition can help researchers and policymakers design and promote innovations that are more likely to be accepted and adopted by farmers (13). By understanding farmers’ perceptions and addressing the factors that influence them, it is possible to accelerate the transition toward more sustainable and environmentally friendly agricultural practices. Understanding farmers’ perceptions and strategies highlights the need to involve multiple actors in co-constructing policies and plans to address challenges in food systems. Additionally, farmers’ perception-centered approach emphasizes the significance of integrating and sharing knowledge from different sources to enhance agricultural productivity and improve the delivery of agricultural extension services to small-scale farmers (14). The literature underscores the importance of stakeholder engagement, innovation management, and entrepreneurship development. It emphasizes the need for a systematic and integrative approach to understand the relationship between these concepts and foster sustainable innovation while considering the interests and concerns of various stakeholders in decision-making processes (15–17).

**Figure 2:**
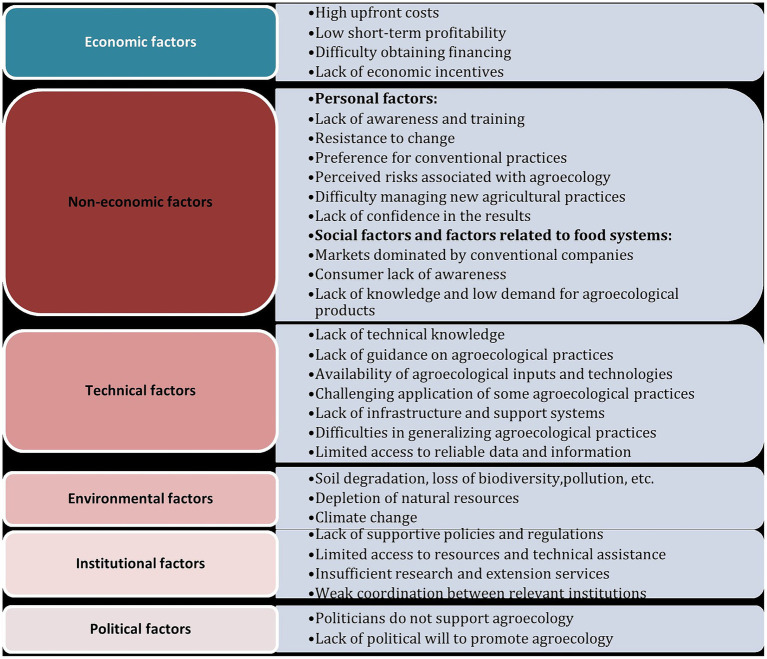
Categories of the factors influencing the agroecological transition.

Another classification of the factors influencing the agroecological transition is revealed according to many studies (Figure 2). These factors are categorized into personal, technical, economic, and social factors. Personal factors pertain to the specific characteristics and beliefs of individual farmers, while technical factors include the knowledge, skills, and resources required for agroecological practices. Economic factors encompass the availability of funds and economic incentives to support the transition. Social factors, on the other hand, are influenced by external factors such as access to grants, markets, and community attitudes (3, 9, 18). These factors are interconnected and can collectively shape the success or barriers to the agroecological transition. Understanding and weighing these factors is crucial when developing strategies to promote sustainable and resilient food systems.

## 3 Methodology

The research methodology is based on a participatory approach supplemented by quantitative and qualitative analysis. The case study is conducted in the northwest region of Tunisia characterized by a mixed tree-crop-livestock farming system.

### 3.1 Study site

**Figure 3:**
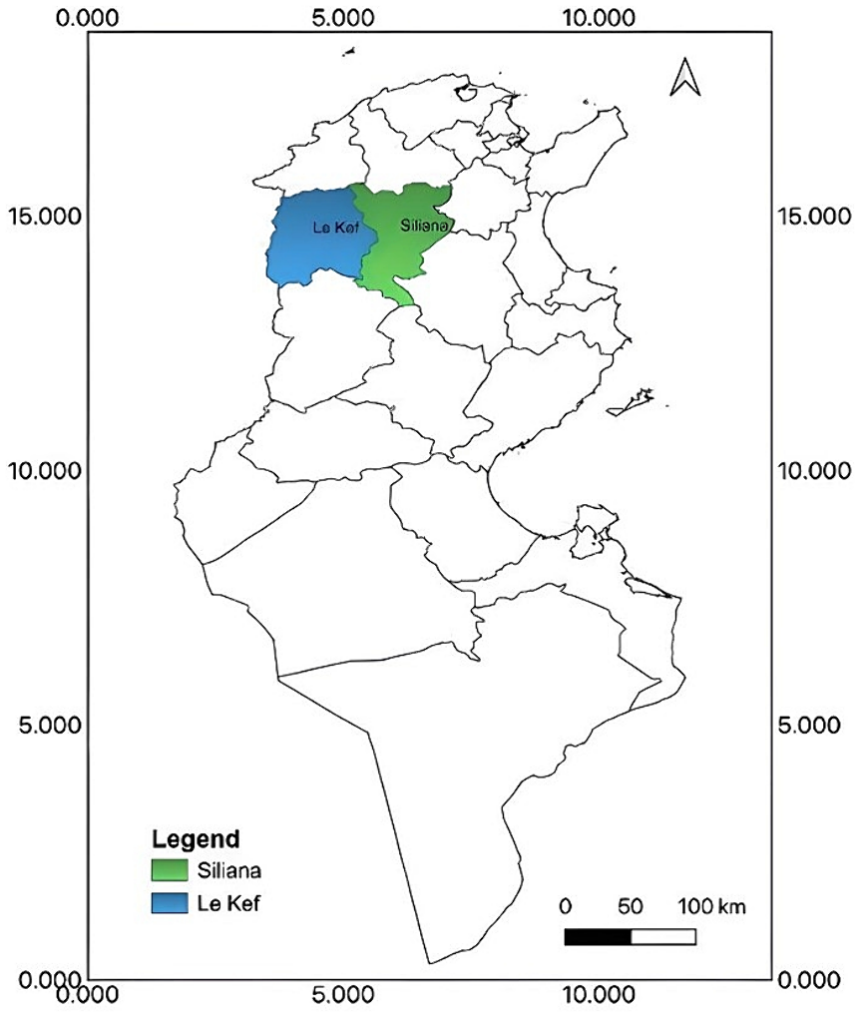
The Tunisian Transect Kef-Siliana localization in the northwest of Tunisia (Source: (19)).

Located in northwest semi-arid zone of Tunisia, the Kef-Siliana transect (Figure 3) has been designated a priority zone by the Agroecology Initiative (19) due to its vulnerability to both soil erosion and climate change (20). While Siliana and Kef governorates both experience a continental climate, their rainfall and temperature ranges differ slightly. Siliana receives between 350 and 550 mm of rain annually with temperatures ranging from 3.2 to 35.7°C, whereas Kef experiences an average annual rainfall of 350 mm to 450 mm and temperatures varying from 7.3 to 26.5°C. These predominantly rural regions face socioeconomic challenges such as high poverty rates, unemployment, and limited access to basic services, leading to significant outmigration, particularly among young people. Despite these challenges, the transect boasts a diversified agricultural system, including cereal crops, livestock farming, and olive tree cultivation. This agricultural diversity reflects the complex interdependence of various sectors and the complexity of the regions’ resource utilization patterns (21, 22).

### 3.2 Data collection

The research involved semi-structured interviews, focus group discussions, workshops, a survey, and a closed-ended questionnaire. The participants were identified based on their expertise, involvement in the initiative, and their roles in the agroecological transition landscape. The selection process has involved reaching out to academic and research institutions, governmental bodies, extension services and other relevant stakeholders to ensure a diverse representation of expertise and perspectives in the study. Table 1 summarizes the different sources of the collected data, the details of the respondents, the research questions, and the methods of analysis. The data were collected in (7) through semi-structured interviews with four professional farming organizations, workshops with farmers, technicians, researchers, public and private stakeholders from various value chains, and an open-ended survey carried out among 69 farmers belonging to farmers’ organizations. Additionally, a questionnaire about the perception of the agroecology transformation barriers and drivers was conducted with 35 farmers engaged in the initiative.

**Table 1:** Overview of data Sources, participants, research questions, and methodology.

Data sources	Respondent details	Research questions	Methods	When used
Four semi-structured interviews and focus group discussion	Four professional organizations of farmers	What is the level of engagement of local communities in developing a specific context for agroecology transformation?	Qualitative analyses, SWOT analysis	May 2022
Two workshops	66 participants: 16 farmers, 14 technicians, 10 researchers, 26 public and private actors from the different identified value chains (notably the olive tree, sheep meat, cereals, honey, and milk)	Which value chains have the most potential to boost the agroecological transition?	An evaluation matrix prioritizing value chains according to agroecological principles	December 2022
A survey	69 Farmers	How do farmers perceive the change toward an agroecological system through project interventions? How does the organizational factor influence the agroecological transformation in the Tunisian context?	Quantitative analyses BBN visualization Descriptive analyses Kendall W test Kruskal-Wallis Test	November 2022
A questionnaire	40 farmers involved in the Agroecology initiative	What are the main boosters and inhibitors of the agroecological transformation considering the current perceptions and characteristics of farmers?	Descriptive analysis Kendall W test Factorial analysis	March 2023

Source: Author’s elaboration (7).

### 3.3 Data sources

The semi-structured interviews and the focus group discussions were conducted with four farmers organizations included in the Tunisian agroecological living landscape in the transect Kef-Siliana. The agroecology initiative is built around the concept and approach of living landscape to integrate the socioeconomic-system and ecosystems in one site to implement and test the agroecological transition (19). The Tunisian living landscape is characterized by the urgent need to enhance natural resource management, foster agricultural innovation, and address climate change impacts effectively. The main objectives of the interviews were to describe the key characteristics of each farmers’ organization and their main activities. To explore the diversity of the key partners and to discuss the main issues/challenges and their propositions to see how the agroecology approach could satisfy their needs.

The workshops were instrumental in identifying the opportunities and challenges to the agroecological transformation and selecting the main value chains with the greatest potential for boosting this transformation. The selection was based on a global evaluation matrix prioritizing the value chains according to a set of predefined criteria based on agroecological principles and their economic, social, and environmental dimensions or criteria (20). These selection criteria are summarized in Table 2. The research by Di Vita et al. (24) and Spina et al. (25) underscores the importance of employing value chain methodologies. Through a holistic approach that involves establishing a focus group with thematic nodes and topics, involving national-level actors and experts, collecting data via interviews, and rigorously processing the gathered information, a comprehensive framework is developed to enhance understanding and decision-making in the field.

**Table 2:** Dimensions for the selection of the value chains.

Economic criteria	Environmental criteria	Social criteria
Job creation opportunities Market demand prospect Income generation Comparative advantage of the production Level of competitiveness	Environmental impact of the value chain Impact of the environment on the value chain	Integration of local actors Improving working conditions

Source: Adapted from Jochem (20).

The survey explores the influences of farmers’ organization on innovative farming practices. It includes questions on the impact of agricultural demonstrations on farmers’ understanding and practices, on trade between farmers, on collective investment, on the perception of the organization of farmers in the community, and on the inclusiveness, exchange of information, commitment, and participation of women within the farmer organization, as well as on contracts and services between the farmer organization and farmers.

The questionnaire on perception is designed based on the factors that were identified in the theoretical framework as influencing the agroecological transformation. It is structured into several sections. The first section focuses on the socio-economic characteristics of the farmers, including age, gender, location, education level, land ownership, main farming activities, and years of experience. The second part of the questionnaire explores the farmers’ perceptions of the agroecological transformation in Tunisia. This section is further divided into four subsections. The first subsection addresses the effects of agroecological practices, the second subsection focuses on the farmers’ capabilities, and the third subsection delves into the difficulties and challenges associated with transformation. The fourth subsection of the questionnaire deals specifically with technical barriers. It is important to note that the active participation in the agroecological transformation was a selection criterion for all the respondents.

### 3.4 Respondents’ characteristics

An overview of the characteristics of the farmers included in the survey and in the questionnaire is included in Table 3. The survey was conducted on a total of 69 farmers, with 38 female and 31 male farmers, while the questionnaire on perception was conducted on 35 farmers, with 6 female and 29 male farmers. The farmers are in Transect Kef-Seliana and Kairouan, with a primary focus on livestock, cereal crops, and olive trees as their main crops.

**Table 3:** Characteristics of the farmers.

	The survey	The questionnaire on perception
Total number of farmers	69	35
Number of female farmers	38	6
Number of male farmers	31	29
Age range (years)	22–73	21–72
Average age (years)	48	51
Location	Transect Kef-Seliana and Kairouan	Transect Kef-Seliana
Main crops	Livestock, cereal crops, olive trees	Olive trees, cereal crops, livestock
Land holdings range (ha)	1–50	0–100
Average land holdings (ha)	9	17

Source: Author’s elaboration from analysis of field data (2023).

The land holdings of the farmers range from 1 to 50 hectares in the survey and 0–100 hectares in the questionnaire, with an average of 9 and 17 hectares, respectively. The age range of the farmers is between 22 and 73 years, with an average age of 48 years for the survey and 51 years for the questionnaire.

### 3.5 Analytical methods

Descriptive statistical analysis was conducted using various basic statistical measures, including mean, standard deviation, maximum, minimum, frequencies, and percentages. In addition, several analytical techniques were employed, such as SWOT analysis, Chi^2^, correlation, Kendall W and Kruskal-Wallis tests, Bayesian Belief Network (BBN) visualization, and factorial analysis. These methods were performed to accomplish several objectives: determining the level of engagement of local communities, prioritizing value chains with high agroecological potential, evaluating the progress toward an agroecological system through project interventions and farmers’ organizations, and assessing and categorizing the different drivers and barriers in the agroecological transformation of the Tunisian food system. The software tools SPSS and Stata were utilized for these analyses.

#### 3.5.1 The SWOT analysis

The SWOT analysis is a strategic tool that helps identify the strengths, weaknesses, opportunities, and threats associated with projects and businesses (26, 27). Its primary purpose is to evaluate both external and internal factors that either support or hinder the progress and successful implementation of projects or programs, aiding in making informed operational decisions (28). This analysis provides a framework for the strategic development of programs or projects, and it has been widely used to explore the internal and external environments, enabling the formulation of strategies and decision-making approaches for projects and programs (29). However, in the context of agroecology research, the SWOT analysis does encounter certain limitations. These limitations encompass subjectivity, the absence of quantifiable metrics hindering precise numerical assessments and comparisons, the dynamic nature of factors necessitating ongoing updates, and the limited focus on interactions, which may not fully consider how different factors in agroecosystems interact and influence each other. This can overlook important connections and complexities within agricultural systems, which are crucial for sustainability and resilience (30, 31). It is crucial to consider these limitations to ensure a comprehensive and balanced evaluation of agroecosystems. Despite the SWOT analysis limitations, it remains relevant in the literature due to its usefulness in exploring possibilities during the decision-making process and its flexibility in combination with other approaches (32–34).

#### 3.5.2 Bayesian belief network

A Bayesian Belief Network (BBN) is a graphical model that represents the probabilistic relationships between different variables. It is a powerful tool for understanding the complex interdependencies among variables and their influence on each other. BBNs are particularly useful for analyzing and visualizing data in fields such as decision analysis, risk assessment, and machine learning (35). In the context of this study, the BBN was used to visualize the relationships between different variables related to perceived changes. It helped to identify and understand how changes in one variable were connected to changes in other variables, providing insights into the overall impact of project interventions.

#### 3.5.3 Factorial analysis

A principal component analysis with a varimax (orthogonal) rotation method is applied to perform exploratory factor analysis. The aim of this analysis was to obtain a factor structure of Agroecological transition perceived drivers and barriers, with both empirical and conceptual support (36). To determine the applicability of factor analysis, Bartlett’s test of sphericity (*p* < 0.05) was used. The number of factors to retain was decided by applying the criteria of eigenvalues greater than 1 (37). Finally, the extracted factors were labeled to give each factor a meaningful definition and meaning for interpretation.

## 4 Results and discussion

### 4.1 Level of engagement of local communities

The general characterization of the four farmers ‘organizations is summarized in Table 4. The farmer organizations have diverse social and technical histories, allowing for the study of agroecological transition dynamics under various social and policy configurations. Farmer Organization 1, established in 2015, focuses on livestock and diverse agricultural production on smaller land holdings, with an exclusively female membership. In contrast, Farmer Organization 4, founded in 2017, specializes in cereal cultivation and livestock farming on larger land areas, boasting a more gender-balanced composition (50% women). Farmer Organization 3, established in 2020, centers its activities around olive trees, fruit trees, and beekeeping on moderate-sized land holdings. Farmer Organization 2, founded in 2022, is primarily involved in livestock farming and cereal crop cultivation on medium-sized land areas, with the lowest number of members and only 11% female representation. These organizations often develop common projects and actions, and their area and number of beneficiaries reflect their radius of action and capacity for scaling out.

**Table 4:** General characterization of the farmers’ organizations.

	Farmer organization 1	Farmer organization 2	Farmer organization 3	Farmer organization 4
Establishment date	2015	2022	2020	2017
Number of members	6	3	3	9
Number of adherents	55	27	114	120
Number of beneficiaries	55	101	240	500
% of women adherents	100%	11%	70%	50%
% less than 35 years old	20%	11%	40%	40%
Main activities	Livestock, Bee keeping, poultry, saffron and vegetable production.	Livestock, cereal crops, olive trees	Fig trees, Olive trees, Cherry trees Beekeeping Cereals	Livestock, cereal crops, olive trees
Land holdings range	2–3 ha	5–20 ha	0.5–5 ha	20–200 ha

Source: Author’s elaboration from analysis of field data (2023).

The SWOT Analysis is performed to assess the agroecological transition potential of the farmer organizations in the transect Kef-Seliana. The findings show that the farmer organizations promote diversified and sustainable farming systems that align with agroecological principles and facilitate a variety of agroecological practices. The key points identified from the SWOT analysis are included in Table 5.

**Table 5:** SWOT analysis assessing the agroecological transition potential of the farmer organizations.

Strengths	Weaknesses
The farmer organizations have clear goals and objectives, such as rural women empowering, promoting sustainable rural development, and facilitating access to inputs for adherents. Farm activity diversification, including cereal crops, beekeeping, livestock breeding, and olive trees, which allows for a variety of agroecological practices. The farmer organizations offer a range of services such as local food artisanal production, commercialization, mechanization, forage seeds distribution, access to inputs, and capacity building.	Challenges and constraints in farm activities, such as the unavailability of seeds and fertilizers, water shortage, small income availability, unavailable and expensive pellets, diseases, marketing issues. The limited membership and adherence to the farmer organizations and limited financial resources and income. Farmer organizations have limited decision-making power.
Opportunity	Threats
Farmer organizations can promote new agroecological practices, such as seed multiplication, direct seeding against erosion, animal feed own production, and by-products recycling. Farmer organizations benefit from partnerships with organizations by receiving technical assistance, equipment, and training. More diversified economic activities and income sources. The increasing number of adherents, especially among young people and women.	Climate change, drought, and soil erosion. Conflicts among extended families and limited decision-making power. Limited market access outside the region, especially for perishable products. Limited access to microfinancing, which hinders farmers’ capacity to invest in new agroecological practices.

Source: Author’s elaboration from analysis of field data (2023).

The interviewed farmer organizations have successful projects and collaborations with various key partners, such as The German International Cooperation (GIZ), The International Center for Agricultural Research in the Dry Areas (ICARDA), and the Regional Agricultural Development Commissariat (CRDA), to access resources, expertise, and funding opportunities. They have implemented various activities, such as local food artisanal production, conservation agriculture practices, crop rotation, forage mixtures (cereal-legumes), mechanization, forage seeds distribution, access to finance, and capacity building, which contribute to environmental and farming sustainability and connectivity. The diversified membership, with a focus on women and young farmers, aligns with the agroecological principle of social equity and justice. While Farmer Organization 1 had 100% female adherents and Farmer Organization 3 had 70% women adherents, Farmer Organization 2 only had 11% women members. Similarly, the percentage of members less than 35 years old varied across the organizations, with Farmer Organization 1 having 20%, Farmer Organization 2 having 11%, Farmer Organization 3 and 4 having 40%. This diversity in gender and age representation highlights that not all farmer organizations in Tunisia exhibit the same level of inclusion of women and young farmers. However, all the studied organizations encourage economic diversity and have a clear purpose in contributing to good governance. According to many studies, farmers’ collectives have different approaches for supporting agroecological transitions, including funding, advice, capacity building, experimentation with new practices, and information exchange (38, 39). Diversified Farming Systems include functional biodiversity in farming practices to maintain ecosystem services like soil fertility, pest and disease control, water use efficiency, and pollination (40). Besides, crop rotation and legumes were identified as the most adequate diversification strategies for intensive rainfed cereal-based cropping systems (41).

### 4.2 High-potential value chains for agroecological transition

**Figure 4:**
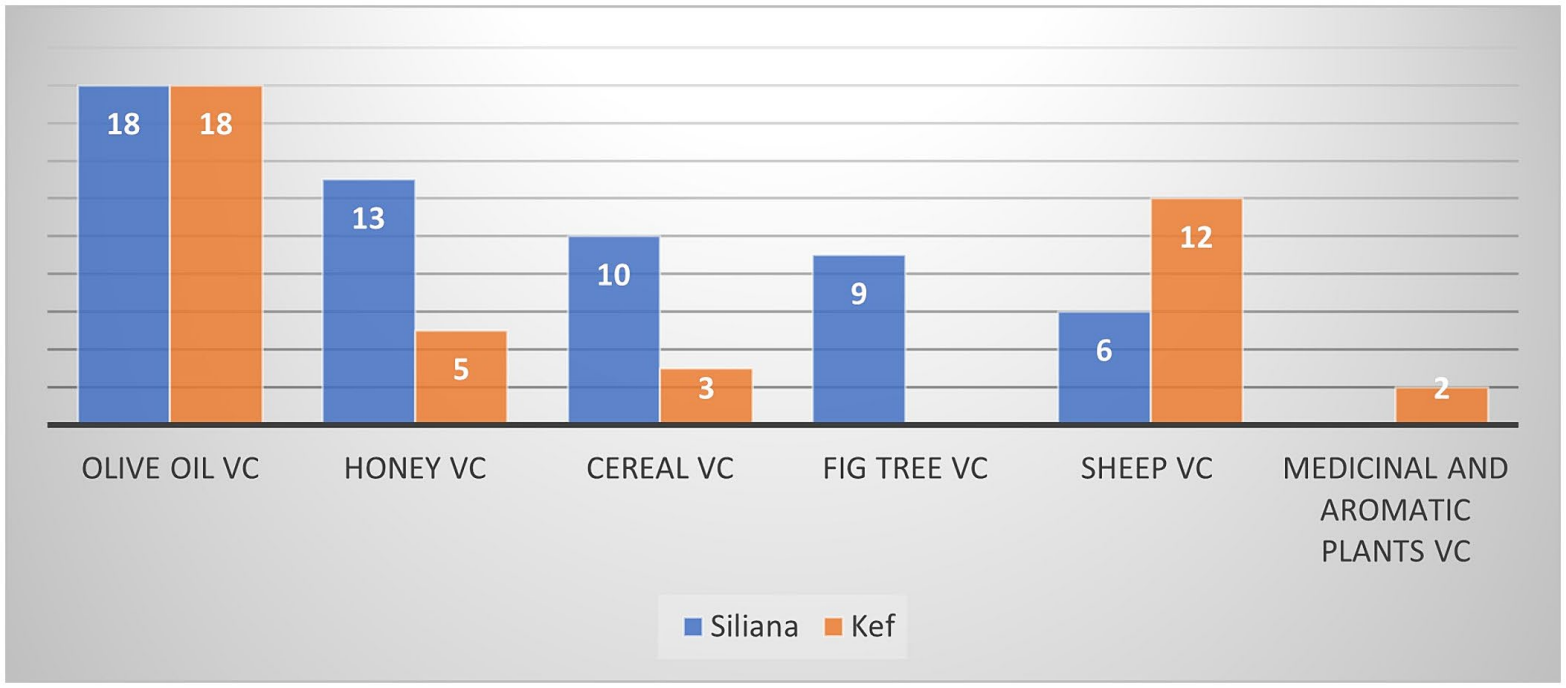
Stakeholder preference for value chains integrated with agroecology principles in Kef and Siliana.

During the workshops conducted with the different stakeholders, many potential value chains were identified including the olive oil, honey, and sheep value chains. Among 33 and 30 participants, respectively in Kef and Siliana, 18 participants in both locations have selected the olive oil value chain as the value chain with the highest potential to integrate agroecology principles, as indicated in Figure 4.

The prioritization of value chains for the agroecological transition in Tunisia highlights the olive oil sector as the most promising for development, considering economic, social, and environmental factors. Table 6 presents the participants motivations regarding the selection of the olive oil value chain.

**Table 6:** Olive oil value chain selection dimensions and arguments.

Value chain selection dimensions	Participant motivations
Economical	*High value creation*: Olive oil production offers significant potential for generating added value, especially through the production of high-quality olive oil. *Expanding industry:* Olive cultivation is experiencing a steady growth in the transect, replacing less profitable crops. *Rising demand and price*: The market for olive oil is expanding due to increasing local and international demand, coupled with an attractive price for producers.
Social	*Cultural significance:* Olive oil is a cherished product, holding deep symbolic value for both consumers and farmers. *Community building:* Olive cultivation strengthens social bonds, particularly during the harvest season (a festive period) and by providing employment opportunities throughout the value chain. *Skilled workforce:* The region boasts a highly skilled workforce of olive farmers who possess extensive knowledge of olive tree cultivation.
Environmental	*Climate-resilient crop:* Olive trees are well-adapted to the changing climate, requiring minimal inputs, energy, and water. Additionally, the olive trees contribute to a balanced ecosystem. *Waste reduction:* Olive cultivation is an eco-friendly practice that utilizes byproducts like olive marc, leaves, and olive tree trunks.

Source: Author’s elaboration from analysis of field data (2023).

### 4.3 Agroecological assessment of the olive oil value chain

The stakeholders present in the workshops were asked if the olive oil value chain can integrate the agroecological principles. The 13 principles of agroecology (42) applied to the selected value chain are presented in Table 7.

**Table 7:** The agroecological principles applied to the olive oil value chain.

Principles	Olive oil value chain
1. Recycling	Wood shredding, using wood as livestock feed, composting, producing charcoal, using olive pomace as livestock feed, utilizing olive water as fertilizers, and using wood in the manufacture of small tools.
2. Input reduction	The olive tree is a low-input crop that can benefit from the use of compost, legume crops as manure, good soil management, and reduced pesticide use.
3. Soil health	Olive plantations help floor fixing and erosion control.
4. Animal health	Olive tree can serve as an animal shelter, it is used as a livestock feed and a source of bee feeding.
5. Biodiversity	Genetic potential in the olive crops. Olive trees can be planted with other trees and can be used as windbreaks to protect other corps.
6. Synergy	There is an ecological interaction between production units, improves water retention capacity, provides food for livestock, water, and soil conservation.
7. Economic diversification	Olive tree provides an income diversification through procuring income in winter, olive is a non-perishable product, and can be sold at any time, by-products can provide additional income, valorization of sub-products improves the farmers’ income, if farmers follow the technical package the productivity will improve.
8. Co-creation of knowledge	Transfer of knowledge, exchange of olive varieties between farmers, co-creation of knowledge can be realized in case the farmers are in an association.
9. Social values and diets	Local product, creation of a label, and high nutritional value.
10. Fairness	Olive oil value chain guarantees decent livelihoods in case there are large areas planted or in case there is intercropping.
11. Connectivity	Sales circuits are short, purchase at the farm, at the oil mill, total lack of connectivity between the institutions in the value chain structures, lack of trust between producer and consumer, an electronic platform on the internet needs to be established.
12. Land and natural resource governance	Institutional support, sector regulation, presence of specialized organizations, and land division due to inheritance.
13. Participation	Limited participation of support organizations (ONH, CRDA, IO, ODESYPANO)^*^, limited involvement in olive variety choices and in decision-making in general.

Source: Author’s elaboration from analysis of field data (2023).*ONH, National Oil Office; CRDA, Regional Agricultural Development Agency; IO, Olive Institute; ODESYPANO Office for the Development of Silvo-Pastoral Systems in the North-West.

Several research studies have backed the views of different stakeholders and considered the multi-stakeholder perspective to identify the obstacles and prospects in the food products’ value chains (24). The goal is to identify potential innovations that align with the needs and perceptions of the stakeholders (16, 43, 44). According to Torquati et al. (45), short extra virgin olive oil supply chains enhance agricultural products’ sustainability, with no real trade-offs when considering value chain results and environmental impact. In the context of the Tunisian olive oil supply chain, an optimal configuration incorporating organic farming, biodynamic growing techniques, and a two-phase extraction system using wet pomace for compost preparation is recommended (46). Circular economy principles can be implemented in the olive oil supply chain, but overcoming technological barriers and knowledge gaps is crucial for advancing circularity in the Mediterranean region’s agroecological systems (47).

### 4.4 Farmers’ perceptions of change

The aim of the survey was to understand how farmers perceive the change toward an agroecological farming system based on project interventions, and what is the influence of the organizational factor in this transformation in the Tunisian context. The descriptive analysis reveals that over 91.3% of farmers who received training and assistance with agroecological practices as part of ICARDA projects reported a change in their ideas and practices, while around 8.7% reported no change at all. These results confirm the findings of Oppong et al. (48), indicating that farmers in Ghana’s semi-deciduous region face challenges in adopting climate-smart agricultural practices due to lack of training, government support and extension officers. According to Šūmane et al. (49) redesigning the farming systems, necessitates farmer engagement in practices and local knowledge production. Integrating researcher and support-oriented strategies to bridge theory and practice is crucial for sustainable agroecological farming systems development (50).

Table 8 illustrates the number and percentage of farmers adopting and not adopting new agroecological practices by age and gender. The project suggests incorporating agroecological practices such as intercropping, direct seeding, minimal tillage, and crop rotation. The total percentage of female respondents is higher than male respondents, with 55 and 45%, respectively. The highest percentage of adopting farmers is in the 41–60 age group, with 36.5% of female respondents and 20.6% of male respondents.

**Table 8:** Farmer’s adoption of agroecological practices by age and gender.

Age of respondents	Non adopting farmers		Adopting farmers		Total respondents	
	Men	Women	Men	Women	Men	Women
20–40	1 (16.6%)	0	11 (17.4%)	6 (9.5%)	12 (17.3%)	6 (8.7%)
41–60	0	4 (66.6%)	13 (20.6%)	23 (36.5%)	13 (18.8%)	27 (39.1%)
61–75	1 (16.6%)	0	5 (8%)	5 (8%)	6 (8.7%)	5 (6.2%)
Total	2 (33.3%)	4 (66.6%)	29 (46%)	34 (54%)	31 (45%)	38 (55%)

Source: Author’s elaboration from analysis of field data (2023).

The Pearson chi-squared (chi^2^) test showed no significant association between location and the adoption of new practices (with a Pearson chi^2^ statistic of 0.3570 and a *p*-value of 0.550) or between gender and the adoption of new practices (Pearson chi^2^ = 0.3570, Pr = 0.550). These results could be explained by the high level of adopting farmers among the respondents. The correlation coefficient between the adoption of new practices and farmer’s age is −0.051, indicating a very weak negative correlation. However, the *p*-value (0.677) suggests that this correlation is not statistically significant. However, many studies reveal that age of farmers have a negative effect on the adoption of sustainable agriculture practices (51–54).

Farmers’ perceptions of the change after research and development projects reveal varying levels of endorsement. In terms of motivation and engagement, change in farming comprehension and practices, and improved information exchange between farmers, these aspects are perceived very positively (Mean = 0.95, 0.92, and 0.91, respectively), indicating strong support for agroecological initiatives (Supplementary Appendix 1). Factors related to inclusiveness of small farmers (Mean = 0.87), participation of women (Mean = 0.78), and commercial exchange between farmers (Mean = 0.70) are viewed more moderately. On the other hand, perception of investment in collective activities (Mean = 0.56) and better services and contracts between the farmers’ organization and agricultural producers (Mean = 0.49) are comparatively lower, suggesting a more nuanced view or potential challenges. Understanding these nuanced perspectives is crucial in tailoring interventions and promoting sustainable agricultural practices.

**Figure 5:**
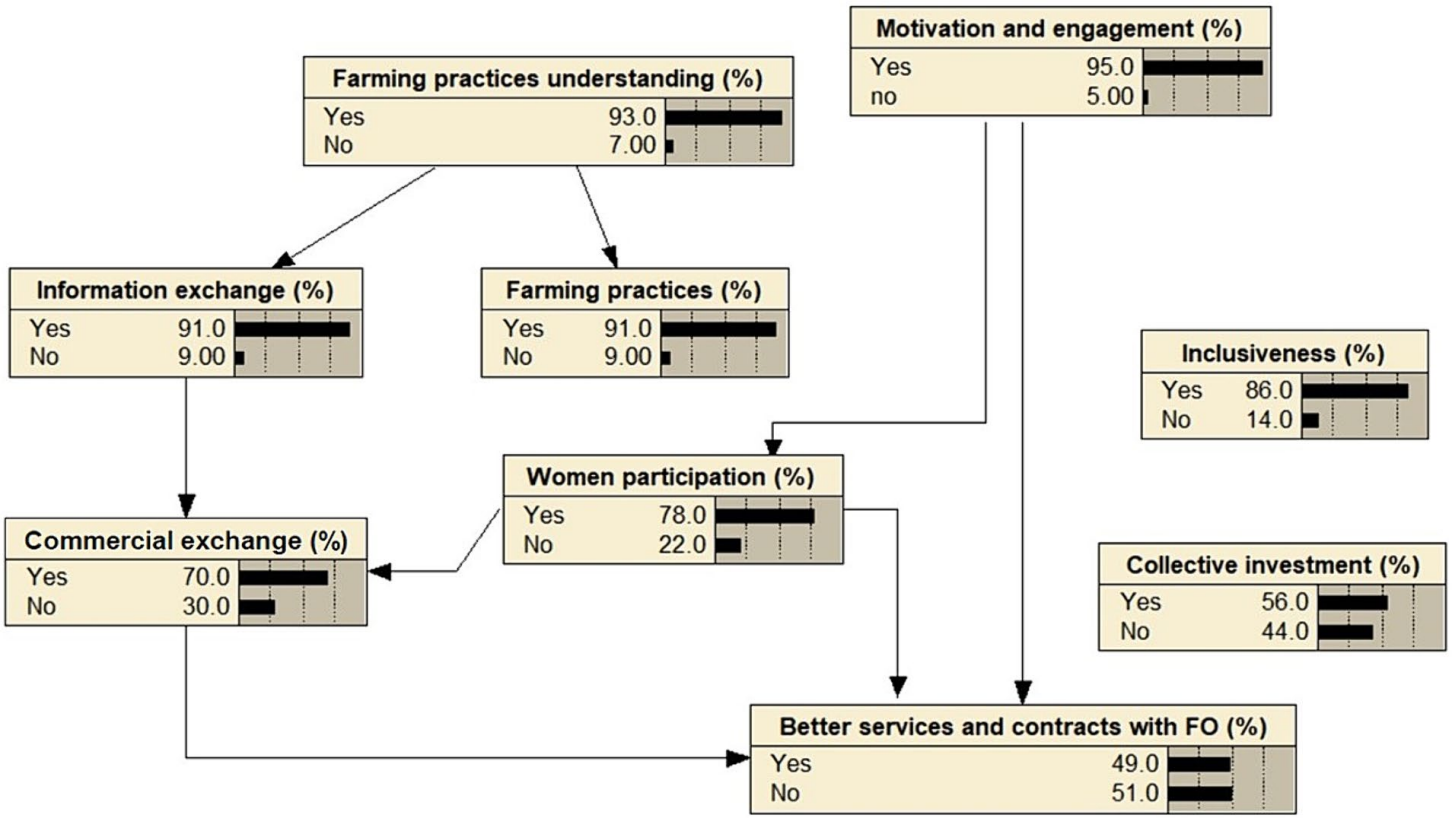
Bayesian Belief Network illustrating the interconnected perceptions of change among farmers.

Through the chi-squared test, statistically significant linkages between various aspects of the perceived change are identified (Supplementary Appendix 2). The visualization via the Bayesian Belief Network (BBN) allows for understanding the complex interdependencies between the different variables (Figure 5). The farmer’s perception of changes in motivation and engagement is linked to the perception of changes in women’s participation and to the enhancement of services and contracts with farmers’ organizations. Likewise, the perception of a better understanding of farming practices is connected to the change of farming practices and to a better information exchange between farmers. Information exchange between farmers is related to the perception of a better commercial exchange that also associated to the enhancement of services and contracts with farm organizations. Only the perception of inclusiveness and collective investments are not connected to other aspects of change. The identification of these interlinks helps prioritizing the intervention areas where interventions had the most significant impact. A higher perceived motivation and engagement suggests the effectiveness of interventions in that domain and may impact women participation and the enhancement of services and contracts with farmers’ organizations. The project’s interventions were also effective leading to a high perceived understanding of farming practices that improves the information exchange between farmers and farming practices change. This insight can guide the design of future interventions based on the identified associations, leading to more targeted and impactful interventions.

**Figure 6:**
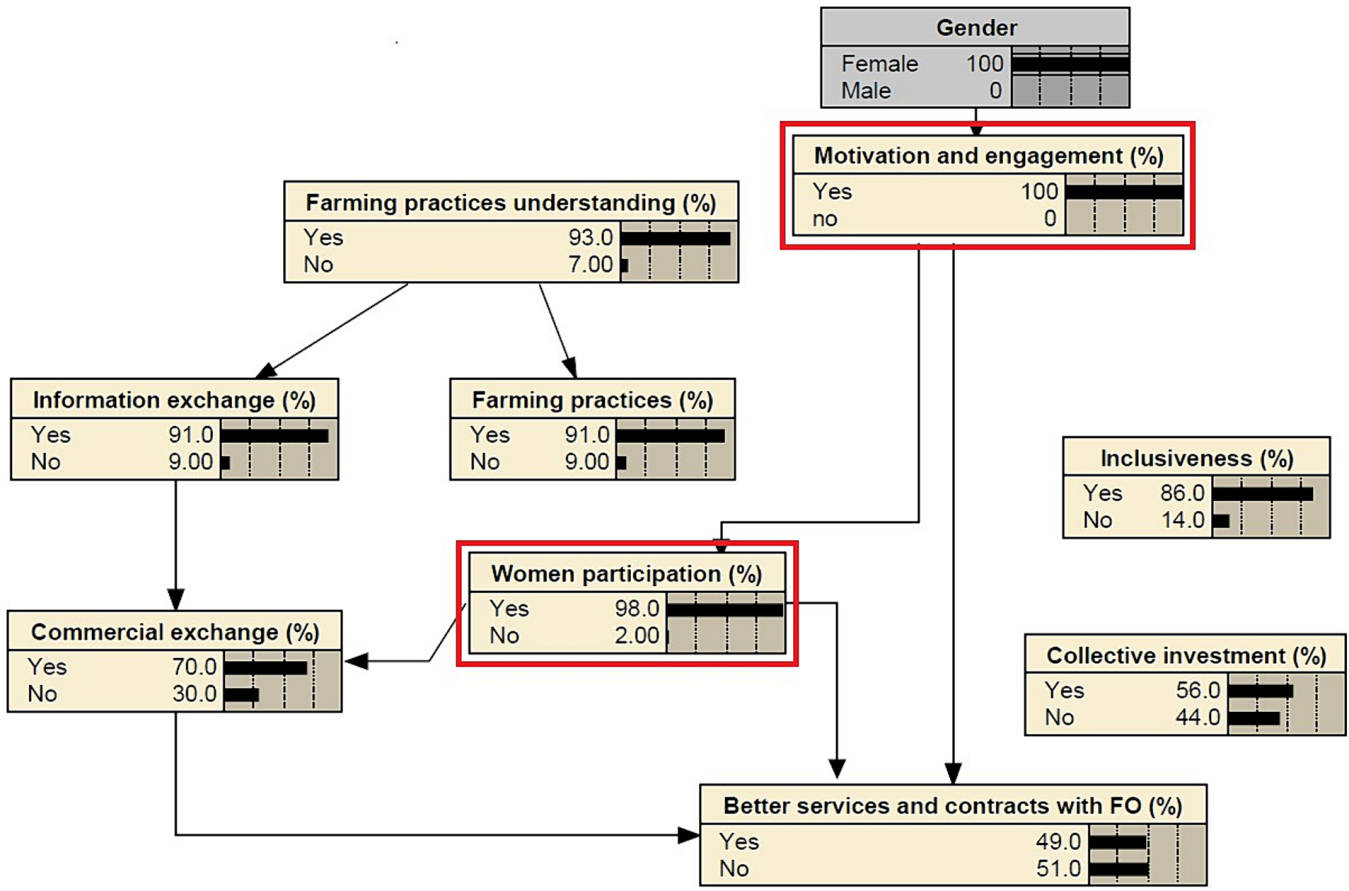
Gender influence in the motivation and engagement perception and in women participation perception of farmers.

The findings suggest a significant association between gender and the perceptions of motivation and engagement in agricultural projects (Supplementary Appendix 3). A strong association is identified between gender and the women’s participation perception and the perception of better services and contracts between farmers and farmer organizations. The study shows that the perception of change on motivation and engagement increases from 95 to 100% if all respondents are women, while the women’s participation perception increases from 78 to 98% (Figure 6). These results are consistent with several studies that have explored the role of gender in agricultural projects. Cloete et al. (55) found that rural Nicaraguan women’s motivations change from initial to sustained forms, enabling them to sustain community-led projects and build social capital, self-efficacy, and agency. Amran and Fatah (56) studied women’s empowerment in agriculture in Malaysia and found that access to extension services and effective decision-making are key factors, but limited leadership, motivation and engagement challenges, and restricted community group participation hinder women’s empowerment. Meinzen-Dick et al. (57) emphasized the importance of integrating gender into agricultural research, development, and extension to enhance food security and promote innovation in developing countries. Gender perceptions can significantly influence smallholder farmers’ adoption of resilient or sustainable farming practices. Studies have shown that women, who are often the most vulnerable smallholder farmers, are bound to benefit from this agricultural technology, mostly because of its attributes (i.e., climate smart practices) (58). Additionally, women have less access to productive resources, financial capital, and advisory services compared to men which may explain women’s high positive perception of motivation, engagement, and participation in projects’ activities (59).

### 4.5 Farmers’ organizations influence in the adoption of innovative farming practices and decision-making change

Table 9 presents the results of farmers’ perceptions of the effects of farmers’ organizations on changing practices and decisions on the farm. The items in the survey included the effect of farmers’ organizations on “changing input purchasing behavior,” “changing practices and techniques for crop management and/or breeding,” “changing sales and marketing behavior,” “changing relationships with other farmers,” and “changing vision for the farm in 10 years.” The results of the reliability analysis using Cronbach’s alpha for a scale composed of the five items show that the average interitem covariance is 1.58, indicating that the items in the scale are positively correlated. The scale reliability coefficient is 0.93, which is considered high and suggests that the scale has good internal consistency. This means that the items in the scale are measuring the same construct and are reliable for measuring that construct.

**Table 9:** Responses on farmer’s perceptions of farmers’ organization effects on changing practices and decisions on the farm (*n* = 69).

Items	SD	D	N	A	SA	Mean	Standard deviation	Decision*
Effect on changing input purchasing behavior	13 (19%)	3 (4%)	9 (13%)	16 (23%)	28 (41%)	3.62	1.51	Low perception
Effect on changing practices and techniques for crop management and/or breeding	14 (20%)	3 (4%)	8 (12%)	14 (20%)	30 (44%)	3.62	1.56	Low perception
Effect on changing sales and marketing behavior	16 (23%)	5 (7%)	8 (12%)	15 (22%)	25 (36%)	3.40	1.59	Low perception
Effect on changing relationship with other farmers	8 (11%)	2 (3%)	6 (9%)	18 (26%)	35 (51%)	4.01	1.33	High perception
Effect on changing your vision for your farm in 10 years	9 (13%)	4 (6%)	7 (10%)	16 (23%)	33 (48%)	3.86	1.41	High perception

SD, strongly disagree; D, disagree; N, neutral; A, agree; SA, strongly agree. *Decision − weighted average = 3.70. Source: Author’s elaboration from analysis of field data (2023).

The weighted average decision score is the sum of the mean values for the five items, divided by the total number of the items. It was 3.70, indicating an overall positive perception of the effects of farmers’ organizations on changing practices and decisions on the farm. The results show that the highest levels of agreement were observed for changing relationships with other farmers and changing the vision of the farm in 10 years. The Kendall W test shows that the five variables presenting the effects of farmers’ organization have similar mean ranks, ranging from 2.69 to 3.27 (Supplementary Appendix 4). This suggests a general agreement that all effects hold some importance. However standard deviations are relatively high, indicating variation in perceived importance among respondents. Kendall’s Coefficient of Concordance (W) was estimated at 0.064 and statistically significant at 10%, indicating a weak level of agreement in the ranking of effects across respondents. The weak concordance suggests individual differences in how they prioritize these effects. There is not a strong consensus on which effect is most or least important.

The findings are consistent with previous studies that highlighted the significance of behavioral, social, and cognitive factors in influencing farmers’ decisions. Spina et al. (60) found that farmers’ attitudes strongly influence their intention to adopt, followed by social norms and perceived control. According to Addai et al. (61), the membership in farmer organizations affects the decision to adopt farm technologies by rice farmers in Ghana. The household head’s decision to adopt new farming practices such as machinery use and row planting increases upon joining a farmer organization. A scoping review of the literature on farmers’ organizations impacts on small-scale producers in sub-Saharan Africa and India found that farmers’ organizations, such as associations, cooperatives, and women’s groups, provide services that contribute to income and productivity for small-scale producers (62). Most reviewed studies reported positive impacts on farmer income, but much fewer reported positive impacts on crop yield and production quality. Environmental benefits, such as resilience-building and improved water quality and quantity, were documented in 24% of the studies. The review suggests that farmers’ organizations could be integrated into policy by having access to markets through information, infrastructure, and logistical support at the center of farmers’ organizations design (62).

To understand if there are any gender disparities in how farmers’ organizations shape farm management, a Kruskal-Wallis’s test was performed. The Kruskal Wallis test is a non-parametric test that compares the medians of two or more groups, and it is used when the data do not meet the assumptions of normality and equal variances required by parametric tests. Results showed that there were significant differences between the two groups in all five variables (*p* < 0.05) (Supplementary Appendix 5). Specifically, women had higher mean ranks than men in the perception of the farmer organization effect on changing input purchasing behavior, on changing practices, on changing sales and marketing behavior, and on changing their vision for the farm in 10 years. The higher mean ranks for the female group suggest they generally perceived these effects as more important than the male farmers. Men had a higher mean rank only in the perception of the farmer organization effect on changing relationship with other farmers. The overall assessment suggests that, on average, females tend to provide higher ratings for the farmer organization effects on changing practices and decisions on the farm compared to males. However, the variability in responses is higher among males, indicating that there might be more diverse opinions among males. Women could be more aware of the farmer organization roles and influences because of the important gap in productivity, income, and resources that women are experiencing. According to Bello et al. (63), a disparity between men and women with a gender performance gap of about 11% in favor of men, is partially explained by factors such as the men access to improved varieties, membership of farmer-based organizations, extension services, and quantity of seeds sown.

Farmers’ organizations play a significant role in influencing the adoption of farming innovative practices and decision-making change. The positive perceptions of the effects of farmers’ organizations on changing practices and decisions on the farm, particularly in relation to changing relationships with other farmers and the long-term vision for the farm, underscore the importance of collaborative and supportive networks in promoting sustainable farming practices. However, the lower levels of agreement regarding changing sales and marketing behavior, as well as input purchasing behavior and crop management practices, suggest that there may be specific areas where farmers’ organizations could focus on enhancing their support and influence.

### 4.6 Farmers perception of agroecological transformation

The findings derived from the perception analysis provide valuable information regarding the farmers’ perception of agroecological transformation drivers and barriers. The descriptive analysis of the sample reveals that most of the participants in the study are male farmers, comprising 83% of the sample. In terms of education level, a significant proportion of the participants have completed secondary education (37%), followed by those with a university level of education (20%). The primary activities of the participants are dominated by olive tree cultivation (43%), with field crop cultivation (28%) and livestock farming (14%) also being prevalent. The participants’ age ranges from 21 to 72 years, with a mean of 52 years. Land ownership among participants varies widely, ranging from no land to 100 hectares, with a mean of 17 hectares. There is only one young farmer (27 years old) who does not own any land. On average, the participants have 28 years of experience as farmers, and their primary activity contributes about 63% of their income, with some variation across individuals (Supplementary Appendix 6).

Respondents’ perceptions about challenges and barriers of adopting agroecological practices are varying from strong agreement to total disagreement. The percentages of respondents for each category, means, standard deviations, decisions, and the ranking of the perceived barriers and motivating factors to the adoption of agroecological practices by farmers are summarized in Supplementary Appendix 7. The highest perceived barriers are the lack of financing and credit opportunities, the lack of encouragement from the government, water shortages, soil erosion, and other environmental problems, the absence of encouraging legislation and laws, the lack of infrastructure and supporting systems, the lack of training on ecological farming, and the lack of production inputs. Improved water conservation and enhanced soil quality are indeed key benefits of the agroecological transition. However, water shortages and soil erosion can still be perceived as barriers due to the initial challenges and adjustments required during the transition process. Despite the eventual benefits, the transition to agroecology may initially pose challenges in adapting to new practices and overcoming existing environmental issues.

Indeed, the most motivating factors perceived by farmers are that agroecological practices contribute to preserving the environment and natural resources, reduce the cost of production, contribute to improved food quality, are compatible with culture and values, contribute to improved production and income, and are compatible with farmers’ knowledge and experience. The most motivating items of the agroecological transformation can be the entry points for the transition. However, the respondents agree less with the facts that agroecological practices and activities are compatible with the financial, economic, technical, and logistical capabilities of farmers. These results are confirmed by Kendall’s W test. The test has been used to assess the level of agreement among respondents’ rankings of various statements related to agroecological practices and their associated motivations, challenges, and barriers. The value of Kendall’s W is 0.20 and the *p*-value is 0.000 (Supplementary Appendix 7). This indicates that there is a statistically significant weak level of agreement among the respondents’ rankings of the various statements related to agroecological practices and their associated challenges and barriers. The mean ranks for each statement provide insight into the relative importance or perception of each item. For example, “Agroecological practices contribute to preserve the environment and natural resources” has the highest mean rank of 21.87, indicating that, on average, respondents ranked this statement as more important or more in agreement compared to other statements. Conversely, “Constraints and complexity of agroecological transition consist of the lack of consumer demand for ecological products” has a lower mean rank of 9.31, indicating that, on average, respondents ranked this statement as less important compared to other statements. The Cronbach’s alpha value is 0.763, indicating an acceptable level of reliability and suggesting a satisfactory level of internal consistency among the items.

### 4.7 Key driver and barrier factors of the agroecological transformation in Tunisia

Factorial analysis is conducted to understand the structure of the main drivers and barriers of the agroecological transformation considering the current perceptions of the Tunisian farmers. The factorial analysis conducted on 30 factors (items) related to agroecological practices reveals a nuanced understanding of the complexities and challenges surrounding their adoption. The analysis delineates 9 key components (explaining 78% of the total variance), each capturing distinct aspects of the agroecological transition process (Supplementary Appendix 8).

- Component 1: captures financial, and economic considerations, alongside logistical and technical feasibility, that emerge as crucial determinants of this first factor labeled as *“Compatibility with farmers’ capabilities and knowledge and capacity building needs.”* This component also focusses on technical difficulties facing ecological transformation, such as the lack of training, technical knowledge, and experience.
- Component 2: highlights key constraints such as the absence of encouraging legislation and laws, and the lack of government support, the delayed results to enhance incomes and the lack of exchange of experiences and of cooperation between farmers. The second factor more related to the perception of barriers is labeled as *“Political, institutional, and communication barriers and risk perception.”*
- Component 3: includes constraints such as the high cost of transition, difficulties in changing production habits and lack of cooperation between the different stakeholders. This factor is labeled as *“Stakeholder cooperation and implementation challenges.”*
- Component 4: emphasizes the alignment of agroecological practices with cultural and economic expectations, including initial productivity changes, cost reduction, and long-term production and income improvement. This component can be interpreted as *“Cultural and economic benefits.”*
- Component 5: highlights logistical difficulties such as input unavailability, the lack of infrastructure and supporting systems and challenges in scaling up agroecological practices. This factor is summarized as a barrier and labeled as *“Logistical difficulties and scaling-up challenges.”*
- Component 6: focuses on environmental aspects, including the contribution of agroecological practices to preserve the environment and natural resources, and constraints related to water shortages, soil erosion, and other environmental problems. This factor can be interpreted as *“Environmental sustainability and mitigation in agroecological practices,”* highlighting the role of environmental challenges and mitigation factors as drivers of the agroecological transformation.
- Component 7: encompasses factors related to access to both economic and non-economic aspects such as access to information, credit, and financial support. This component considered as *a barrier* and is identified as *“Access to information and financial services.”*
- Component 8: highlights constraints such as the lack of consumer demand for ecological products, marketing difficulties, and market access challenges. The component is identified as *“Market-related factors.”*
- Component 9: suggests that agroecological practices contribute to improved food quality and hygiene and can be interpreted as *“Health Determinants”* factor.

**Figure 7:**
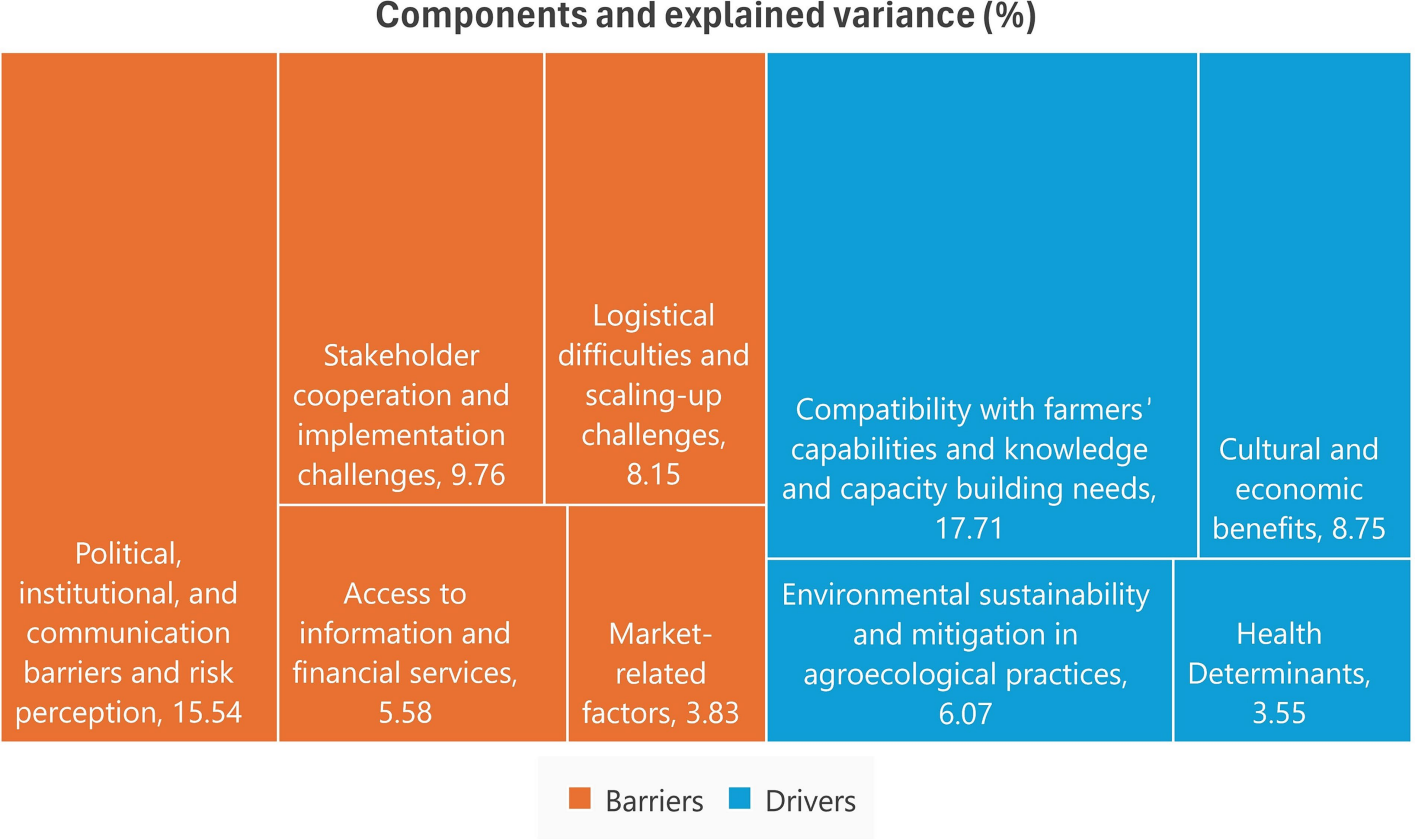
Key drivers and barriers of agroecological transition.

Figure 7 presents drivers and barriers in agroecological transitions in Tunisia according to the local farmers involved. The total explained variance by the extracted components reached 78%. The drivers include compatibility with farmers’ capabilities (17.71%), cultural and economic benefits (8.75%), environmental sustainability (6.07%), and health determinants (3.55%). Political, institutional, and communication barriers (15.54%), stakeholder cooperation challenges (9.76%), logistical difficulties (8.15%), access to information and financial services (5.58%), and market-related factors (3.83%) are identified as barriers. Consistent with these results, the literature highlights the complexity of the factors involved as barriers of agroecological transitions (64). Furthermore, the sustainability of transitions to agroecology is linked to factors such as capacity building, social capital, and farmer knowledge, emphasizing the multifaceted nature of these transitions (65).

## 5 Conclusion and implications

In examining the potential for agroecological transitions in Tunisia, specifically the Kef-Siliana transect, this study has revealed valuable insights. The SWOT analysis demonstrates that farmer organizations have clear goals, diversified farming systems, and partnerships in collaboration with various organizations and institutions. The study emphasizes the significant potential of these farmers’ organizations in advancing sustainable farming practices. However, it also underscores the need for targeted efforts to address specific challenges in farming practices and decision-making. Outlined obstacles include the unavailability of seeds and fertilizers, water shortage, limited income, diseases, and marketing issues. To prioritize value chains for agroecological transition in Tunisia, the study identifies the olive oil sector as the most promising for development, considering economic, social, and environmental factors. Implementing recycling and input minimization principles in the olive oil supply chain and bridging the gap between theoretical agroecological concepts and farming practice implementation are recommended to cultivate sustainable agroecological farming systems. The survey’s results indicate that farmers who received training and assistance with agroecological practices reported positive changes in their ideas and practices. Therefore, the study emphasizes the importance of farmer engagement, knowledge production, and multi-stakeholder collaboration in promoting agroecological transitions in Tunisia. The Bayesian Belief Network (BBN) visualization highlights complex interdependencies between different factors, emphasizing the significance of women’s participation, improved services and contracts with farmers’ organizations, and a better understanding of farming practices to facilitate agroecological transitions. The study identifies various challenges and barriers, including political, institutional, and communication barriers, logistical difficulties, and market-related factors. To address these challenges and facilitate agroecological transitions, the study emphasizes the need for farmer engagement, knowledge production, and multi-stakeholder collaboration. Furthermore, it suggests targeted efforts to address specific aspects of farming practices and decision-making. The study’s findings also underscore the influence of gender perceptions on the adoption of resilient and sustainable farming practices among smallholder farmers, emphasizing the importance of integrating gender into agricultural research, development, and extension to enhance food security and foster innovation in Tunisia. At the political and institutional level, the study recommends the increase of public incentives and supportive legislation to support agroecological practices. Additionally, the study suggests offering innovative financing and credit opportunities to farmers to overcome the lack of production inputs and limited access to microfinancing. Recognizing the lack of training on ecological farming as a significant barrier, the study proposes the development of capacity building programs to equip farmers with the necessary knowledge and skills to embrace agroecological practices.

## Data availability statement

The raw data supporting the conclusions of this article will be made available by the authors, without undue reservation.

## Ethics statement

Ethical approval was not required for the studies involving humans because the farmers participated voluntarily and provided their consent to answer the survey questions. The studies were conducted in accordance with the local legislation and institutional requirements. The participants provided their written informed consent to participate in this study.

## Author contributions

AS: Conceptualization, Formal analysis, Methodology, Writing – original draft, Data curation, Investigation, Software. BD: Conceptualization, Funding acquisition, Methodology, Supervision, Validation, Writing – review & editing, Project administration. AO: Validation, Writing – review & editing. RM: Data curation, Investigation, Writing – original draft. AF: Funding acquisition, Project administration, Resources, Writing – review & editing. MZ: Investigation, Writing – review & editing. MD: Investigation, Writing – review & editing.

## Footnotes

^1^More about this initiative can be read here: https://www.cgiar.org/initiative/agroecology/.
