# Supplementary material for: Linking farmers’ perceptions and management decision toward sustainable agroecological transition: evidence from rural Tunisia
Source: Front Nutr. 2024 May 13;11:1389007. doi: 10.3389/fnut.2024.1389007 (PMC11128687; doi:10.3389/fnut.2024.1389007)
Supplement: Supplementary file 1 [file Table_1.DOCX]

Supplementary material: Appendix tables

**Appendix 1.** Change perceptions variables overview (n=69)

| **Variable** | **Variable label** | **Mean** | **Standard deviation** |
| --- | --- | --- | --- |
| **Understanding** | Understanding farming practices | 0.93 | 0.26 |
| **Farming** | Farming practices | 0.91 | 0.28 |
| **Com-Exchange** | Commercial exchange between farmers | 0.70 | 0.46 |
| **Investment** | Collective investment | 0.57 | 0.50 |
| **Inclusiveness** | Inclusiveness | 0.87 | 0.34 |
| **Info-exchange** | Information exchange | 0.91 | 0.28 |
| **Motivation** | Motivation and engagement | 0.96 | 0.21 |
| **Participation** | Women participation | 0.78 | 0.42 |
| **Services** | Better services and contracts with farmers’ organization | 0.49 | 0.50 |

Source: Author’s elaboration from analysis of field data (2023).

**Appendix 2.** Chi-square associations between perceptions of different aspects of change (n = 69)

| Variable | Understanding | Farming | Com-Exchange | Investment | Inclusiveness | Info-exchange | | Motivation | Participation | Services |
| --- | --- | --- | --- | --- | --- | --- | --- | --- | --- | --- |
| Understanding | ---- | 56.601  (0.000) *** | 2.225  (0.136) | 0.598  (0.439) | 0.230  (0.632) | | 17.871  (0.000) *** | 0.245  (0.621) | 0.009  (0.922) | 1.848  (0.174) |
| Farming |  | --- | 1.188 (0.276) | 1.437  (0.230) | 0.0761  (0.783) | | 14.120  (0.000) *** | 0.298  (0.585) | 0.099  (0.753) | 2.795  (0.095) * |
| Com-Exchange |  |  | --- | 0.210  (0.646) | 0.9595  (0.327) | | 8.685  (0.003) *** | 1.944  (0.163) | 7.913  (0.005) *** | 7.832  (0.005) *** |
| Investment |  |  |  | --- | 0.003  (0.950) | | 1.437  (0.230) | 0.131  (0.717) | 0.079  (0.778) | 0.749  (0.387) |
| Inclusiveness |  |  |  |  | --- | | 2.385  (0.122) | 0.470  (0.493) | 0.817  (0.366) | 0.096  (0.756) |
| Info-exchange |  |  |  |  |  | | --- | 2.397  (0.121) | 0.519  (0.471) | 0.668  (0.414) |
| Motivation |  |  |  |  |  | |  | --- | 3.721  (0.054) * | 3.046  (0.081) * |
| Participation |  |  |  |  |  | |  |  | --- | 6.572  (0.010) ** |
| Services |  |  |  |  |  | |  |  |  | --- |

Pearson chi^2^, (p-value), ***Significance level at 1%, ** Significance level at 5%, * significance level at 10%

Source: Author’s elaboration from analysis of field data (2023).

**Appendix 3.** Gender and perceptions of change after project interventions.

|  | **Positive perception** | | **Negative perception** | |  |
| --- | --- | --- | --- | --- | --- |
| **Variable** | Male (n=31) | Female (n=38) | Male (n=31) | Female (n=38) | Chi^2^ |
| **Understanding** | 29 | 35 | 2 | 3 | 0.053 |
| **Farming** | 29 | 34 | 2 | 4 | 0.357 |
| **Com-Exchange** | 18 | 30 | 13 | 8 | 3.517* |
| **Investment** | 18 | 21 | 13 | 17 | 0.055 |
| **Inclusiveness** | 26 | 34 | 5 | 4 | 0.473 |
| **Info-exchange** | 28 | 35 | 3 | 3 | 0.068 |
| **Motivation** | 28 | 38 | 3 | 0 | 3.845* |
| **Participation** | 17 | 37 | 14 | 1 | 18.151*** |
| **Services** | 9 | 25 | 22 | 13 | 9.229*** |

Pearson chi^2^, (p-value), ***Significance level at 1%, ** Significance level at 5%, * significance level at 10%

Source: Author’s elaboration from analysis of field data (2023).

**Appendix 4.** Kendall's W Test

| **Variable** | **Variable label** | **Mean Rank** | **Ranks** |
| --- | --- | --- | --- |
| Input-purchasing | Effect on changing Input purchasing behavior | 2.93 | **4** |
| Practices | Effect on changing practices and techniques for crop management/ breeding | 2.99 | **3** |
| Marketing | Effect on changing sales and marketing behavior | 2.69 | **5** |
| Relationships | Effect on changing relationship with other farmers | 3.27 | **1** |
| Vison | Effect on changing your vision for your farm in 10 years | 3.13 | **2** |
|  | **N** | | 69 |
|  | **Kendall's W^a^** | | 0.064 |
|  | **Chi-Square** | | 17.79 |
|  | **df** | | 4 |
|  | **Asymp. Sig.** | | 0.001 |

**Note:** The importance of the various effects of farmer organizations was assessed through a ranking scheme developed with 1 indicating minimal importance and 5 signifying maximal importance.

**a.** Kendall's Coefficient of Concordance.

Source: Author’s elaboration from analysis of field data (2023).

**Appendix 5.** Kruskal-Wallis Test

| **Ranks** | | | | | | | | |
| --- | --- | --- | --- | --- | --- | --- | --- | --- |
|  | | **Genre** | | **N** | | | **Mean rank** | |
| Input-purchasing | | Women | | 38 | | | 42.33 | |
|  |  | Men | | 31 | | | 26.02 | |
|  |  | Total | | 69 | | |  | |
| Practices | | Women | | 38 | | | 40.87 | |
|  |  | Men | | 31 | | | 27.81 | |
|  |  | Total | | 69 | | |  | |
| Marketing | | Women | | 38 | | | 41.76 | |
|  |  | Men | | 31 | | | 26.71 | |
|  |  | Total | | 69 | | |  | |
| Relationships | | Women | | 38 | | | 39.42 | |
|  |  | Men | | 31 | | | 29.58 | |
|  |  | Total | | 69 | | |  | |
| Vision | | Women | | 38 | | | 39.75 | |
|  |  | Men | | 31 | | | 29.18 | |
|  |  | Total | | 69 | | |  | |
| **Statistics Test ^a,b^** | | | | | | | | |
|  | Input-purchasing | | Practices | | Marketing | Relationships | | Vision |
| **Chi-Square** | 12.378 | | 8.044 | | 10.358 | 4.834 | | 5.419 |
| **df** | 1 | | 1 | | 1 | 1 | | 1 |
| **Asymp. Sig.** | 0.000 | | 0.005 | | 0.001 | 0.028 | | 0.020 |
| **a.** Kruskal Wallis test | | | | | | | | |
| **b.** Grouping Variable: Gender | | | | | | | | |

Source: Author’s elaboration from analysis of field data (2023).

**Appendix 6.** Descriptive statistics of the sample

|  | **Frequency** | **Percentage (%)** |
| --- | --- | --- |
| **Gender**  Male  Female | 29  6 | 83  17 |
| **Education level**  Illiterate  Primary  Secondary  University | 1  14  13  7 | 3  4  37  20 |
| **First main activity**  Livestock  Field crops  Olive trees  Beekeeping  Vegetables | 5  10  15  3  2 | 14  28  43  9  6 |

|  | **N** | **Minimum** | **Maximum** | **Mean** | **Standard deviation** |
| --- | --- | --- | --- | --- | --- |
| **Age** | 35 | 21 | 72 | 51.54 | 11.61 |
| **Owned land (ha)** | 35 | 0 | 100 | 17.68 | 20.97 |
| **Years of experience as a farmer** | 32 | 5 | 50 | 28.41 | 11.72 |
| **Percentage of income (first main activity)** | 34 | 15 | 100 | 62.65 | 17.85 |

Source: Author’s elaboration from analysis of field data (2023).

**Appendix 7.** Respondents’ perceptions about challenges and barriers of adopting agroecological practices and Kendall's W Test (n=35)

| Items | Strongly disagree (%) | Disagree (%) | Neutral (%) | Agree (%) | Strongly agree (%) | Mean | Standard deviation | Decision* | Mean Rank | Rank |
| --- | --- | --- | --- | --- | --- | --- | --- | --- | --- | --- |
| Agroecological practices contribute to improved production and income | 2.9 | 5.7 | 2.9 | 34.3 | 54.3 | 4.31 | 0.993 | High perception | 17.57 | 9 |
| Agroecological practices contribute to preserve the environment and natural resources | 0 | 0 | 2.9 | 8.6 | 88.6 | 4.86 | 0.430 | High perception | 21.87 | 1 |
| Agroecological practices reduce the cost of production | 0 | 2.9 | 8.6 | 31.4 | 57.1 | 4.43 | 0.778 | High perception | 18.89 | 5 |
| Agroecological practices contribute to improved food quality | 8.6 | 2.9 | 0 | 20 | 68.6 | 4.37 | 1.215 | High perception | 19.06 | 7 |
| Agroecological practices contribute to decline productivity in the first years | 8.6 | 0 | 34.3 | 25.7 | 31.4 | 3.71 | 1.178 | Low perception | 13.11 | 19 |
| Agroecological practices and activities are compatible with your technical capabilities | 20 | 8 | 17.1 | 34.3 | 20 | 3.26 | 1.421 | Low perception | 11.27 | 25 |
| Agroecological practices and activities are compatible with your culture and values | 0 | 5.7 | 11.4 | 25.7 | 57.1 | 4.34 | 0.906 | High perception | 17.89 | 8 |
| Agroecological practices and activities are compatible with your knowledge and experience | 0 | 0 | 11.4 | 51.4 | 37.1 | 4.26 | 0.657 | High perception | 16.14 | 11 |
| Agroecological practices and activities are compatible with your financial and economic capabilities | 17.1 | 14.3 | 8.6 | 28.6 | 31.4 | 3.43 | 1.501 | Low perception | 13.16 | 23 |
| Agroecological practices and activities are compatible with your logistical capabilities | 25.7 | 14.3 | 22.9 | 17.1 | 20 | 2.91 | 1.483 | Low perception | 9.61 | 29 |
| Constraints and complexity of agroecological transition consist on the difficulty of applying and experimenting agroecological practices | 28.6 | 14.3 | 11.4 | 22.9 | 22.9 | 2.97 | 1.581 | Low perception | 10.29 | 27 |
| Constraints and complexity of agroecological transition consist of the difficulty of changing your production habits | 28.6 | 14.3 | 17.1 | 14.3 | 25.7 | 2.94 | 1.589 | Low perception | 10.17 | 28 |
| Constraints and complexity of agroecological transition consist on the lack of cooperation between actors | 14.3 | 2.9 | 8.6 | 17.1 | 57.1 | 4 | 1.455 | High perception | 16.30 | 16 |
| Constraints and complexity of agroecological transition consist of the lack of exchange of experiences and of cooperation between farmers | 22.9 | 11.4 | 5.7 | 17.1 | 42.9 | 3.46 | 1.669 | Low perception | 13.86 | 21 |
| Constraints and complexity of agroecological transition consist of the lack of encouragement from the government | 2.9 | 5.7 | 2.9 | 14.3 | 74.3 | 4.51 | 1.011 | High perception | 19.47 | 3 |
| Constraints and complexity of agroecological transition consist of the absence of encouraging legislation and laws | 2.9 | 0 | 20 | 5.7 | 71.4 | 4.43 | 1.008 | High perception | 18.67 | 6 |
| Constraints and complexity of agroecological transition consist of the impact of pesticide and fertilizer companies and major food industries | 2.9 | 5.7 | 17.1 | 22.9 | 51.4 | 4.14 | 1.089 | High perception | 16.59 | 14 |
| Constraints and complexity of agroecological transition consist of the lack of financing and credit opportunities in agroecology | 2.9 | 2.9 | 2.9 | 17.1 | 74.3 | 4.57 | 0.917 | High perception | 20.16 | 2 |
| Constraints and complexity of agroecological transition consist of the high cost of transition | 22.9 | 11.4 | 5.7 | 20 | 40 | 3.43 | 1.65 | Low perception | 13.47 | 22 |
| Constraints and complexity of agroecological transition consist of the lack of production inputs | 8.6 | 5.7 | 5.7 | 22.9 | 57.1 | 4.14 | 1.287 | High perception | 16.93 | 13 |
| Constraints and complexity of agroecological transition consist of marketing and market access difficulties | 22.9 | 22.9 | 11.4 | 11.4 | 31.4 | 3.06 | 1608 | Low perception | 11.61 | 26 |
| Constraints and complexity of agroecological transition consist of the lack of consumer demand for ecological products | 31.4 | 22.9 | 11.4 | 17.1 | 17.1 | 2.66 | 1.514 | Low perception | 9.31 | 30 |
| Constraints and complexity of agroecological transition consist of the delayed results to enhance incomes | 11.4 | 5.7 | 17.1 | 34.3 | 31.4 | 3.69 | 1.301 | Low perception | 12.86 | 20 |
| Constraints and complexity of agroecological transition consist of the water shortages, soil erosion and other environmental problems | 2.9 | 5.7 | 11.4 | 2.9 | 77.1 | 4.46 | 1.094 | High perception | 19.39 | 4 |
| The most important technical difficulties facing ecological transformation are: the lack of technical knowledge and guidance on agroecological practices | 8.6 | 14.3 | 5.7 | 14.3 | 57.1 | 3.97 | 1.424 | High perception | 17.16 | 17 |
| The most important technical difficulties facing ecological transformation are: the lack of formative courses and training on ecological farming | 5.7 | 11.4 | 5.7 | 11.4 | 65.7 | 4.2 | 1.302 | High perception | 18.47 | 12 |
| The most important technical difficulties facing ecological transformation are: the challenges in implementing complex agroecological practices | 8.6 | 22.9 | 11.4 | 40 | 17.1 | 3.34 | 1.259 | Low perception | 11.73 | 24 |
| The most important technical difficulties facing ecological transformation are: the limited access to reliable data and information | 8.6 | 8.6 | 5.7 | 25.7 | 51.4 | 4.03 | 1.317 | High perception | 16.41 | 15 |
| The most important technical difficulties facing ecological transformation are: the lack of infrastructure and supporting systems | 5.7 | 2.9 | 11.4 | 14.3 | 65.7 | 4.31 | 1.157 | High perception | 18.51 | 10 |
| The most important technical difficulties facing ecological transformation are: difficulties in scaling up agroecological practices | 17.1 | 5.7 | 8.6 | 22.9 | 45.7 | 3.74 | 1.521 | Low perception | 15.07 | 18 |
| N | | | | | | | | 35 | | |
| Kendall's W^a^ | | | | | | | | 0.202 | | |
| Chi-Square | | | | | | | | 204.554 | | |
| df | | | | | | | | 29 | | |
| Asymp. Sig. | | | | | | | | 0.000 | | |

*Decision - weighted average = **3.86.**

**a.** Kendall's Coefficient of Concordance.

Source: Author’s elaboration from analysis of field data (2023).

**Appendix 8.** Rotation of the component matrix

| Items | Component | | | | | | | | |
| --- | --- | --- | --- | --- | --- | --- | --- | --- | --- |
|  | 1 | 2 | 3 | 4 | 5 | 6 | 7 | 8 | 9 |
| Agroecological practices and activities are compatible with your financial and economic capabilities | **0.882** |  |  |  |  |  |  |  |  |
| Agroecological practices and activities are compatible with your logistical capabilities (transport and transportation of goods and raw materials) | **0.851** |  |  |  |  |  |  |  |  |
| Agroecological practices and activities are compatible with your technical capabilities | **0.759** |  | -0.265 |  | 0.289 |  |  |  | 0.243 |
| The most important technical difficulties facing ecological transformation are the lack of formative courses and training on ecological farming | **0.563** |  | 0.481 | 0.292 | -0.236 |  | -0.240 |  |  |
| The most important technical difficulties facing ecological transformation are the lack of technical knowledge and guidance on agroecological practices | **0.533** |  | 0.333 | 0.517 |  |  | -0.275 |  | 0.252 |
| Agroecological practices and activities are compatible with your knowledge and experience | **0.477** |  | -0.372 | -0.277 | -0.258 | 0.366 | -0.251 |  | -0.210 |
| Constraints and complexity of agroecological transition consist of the absence of encouraging legislation and laws |  | **0.895** |  |  |  |  |  |  |  |
| Constraints and complexity of agroecological transition consist of the lack of encouragement from the government |  | **0.873** |  |  |  |  |  |  |  |
| Constraints and complexity of agroecological transition consist of the impact of pesticide and fertilizer companies and major food industries |  | **0.541** | -0.369 |  | 0.367 |  |  | -0.211 | 0.317 |
| Constraints and complexity of agroecological transition consist of the delayed results to enhance incomes |  | **0.532** |  | -0.343 |  |  | 0.476 |  | -0.401 |
| Constraints and complexity of agroecological transition consist of the lack of exchange of experiences and of cooperation between farmers |  | **0.522** | 0.429 | 0.245 | 0.333 | 0.315 |  |  |  |
| Constraints and complexity of agroecological transition consist of the difficulty of applying and experimenting agroecological practices |  |  | **0.879** |  |  |  |  |  |  |
| Constraints and complexity of agroecological transition consist of the difficulty of changing your production habits |  | 0.278 | **0.817** |  |  |  |  |  |  |
| Constraints and complexity of agroecological transition consist of the lack of cooperation between actors: middlemen, industrialists, merchants, and state institutions |  | 0.423 | **0.534** |  |  | 0.463 |  |  |  |
| Constraints and complexity of agroecological transition consist of the high cost of transition | -0.210 | -0.251 | **0.442** | -0.306 |  |  | 0.427 |  | -0.227 |
| Agroecological practices contribute to decline productivity in the first years |  |  |  | **-0.767** |  |  | 0.270 |  |  |
| Agroecological practices reduce the cost of production | 0.355 | 0.358 |  | **0.684** |  |  |  |  |  |
| Agroecological practices contribute to improved production and income | 0.342 | -0.206 |  | **0.664** |  |  |  |  | 0.292 |
| Agroecological practices and activities are compatible with your culture and values | 0.295 |  | -0.367 | **-0.514** | -0.217 | 0.311 |  | 0.241 |  |
| The most important technical difficulties facing ecological transformation are the challenges in implementing complex agroecological practices |  |  |  |  | **0.779** |  |  |  |  |
| Constraints and complexity of agroecological transition consist of the lack of production inputs (fertilizers, seeds...), equipment and techniques of agroecology |  |  |  | 0.301 | **0.738** |  |  |  | -0.223 |
| The most important technical difficulties facing ecological transformation are difficulties in scaling up agroecological practices | -0.253 |  |  |  | **0.716** |  |  | 0.330 | 0.313 |
| The most important technical difficulties facing ecological transformation are the lack of infrastructure and supporting systems |  |  |  | -0.234 | **0.585** | 0.447 | 0.447 |  |  |
| Constraints and complexity of agroecological transition consist of the lack of financing and credit opportunities in agroecology | -0.259 |  |  |  |  | **0.851** |  |  |  |
| The most important technical difficulties facing ecological transformation are the limited access to reliable data and information |  |  |  |  |  | **0.850** |  |  |  |
| Agroecological practices contribute to preserve the environment and natural resources |  |  |  |  |  |  | **0.831** |  |  |
| Constraints and complexity of agroecological transition consist of the water shortages, soil erosion and other environmental problems |  |  |  |  |  | 0.462 | **0.757** |  |  |
| Constraints and complexity of agroecological transition consist of the lack of consumer demand for ecological products |  |  |  | -0.221 |  |  |  | **0.870** |  |
| Constraints and complexity of agroecological transition consist of marketing and market access difficulties |  | 0.245 | 0.267 | 0.233 | 0.377 |  |  | **0.687** |  |
| Agroecological practices contribute to improved food quality | 0.411 |  |  |  |  |  |  |  | **0.787** |

Extraction method: Principal Component Analysis; Rotation method: Varimax with Kaiser normalization

a. Convergence of the rotation in 17 iterations.

Source: Author’s elaboration from analysis of field data (2023).
